# Effects of High Temperature Frying of Spinach Leaves in Sunflower Oil on Carotenoids, Chlorophylls, and Tocopherol Composition

**DOI:** 10.3389/fchem.2017.00019

**Published:** 2017-03-22

**Authors:** Alam Zeb, Parveen Nisar

**Affiliations:** Biochemistry Laboratory, Department of Biotechnology, Faculty of Biological Sciences, University of MalakandChakdara, Pakistan

**Keywords:** carotenoids, chlorophylls, spinach leaves, frying, HPLC-DAD, leafy vegetables

## Abstract

Spinach is one of the highly consumed vegetable, with significant nutritional, and beneficial properties. This study revealed for the first time, the effects of high temperature frying on the carotenoids, chlorophylls, and tocopherol contents of spinach leaves. Spinach leaves were thermally processed in the sunflower oil for 15, 30, 45, and 60 min at 250°C. Reversed phase HPLC-DAD results revealed a total of eight carotenoids, four chlorophylls and α-tocopherol in the spinach leaves. Lutein, neoxanthin, violaxanthin, and β-carotene-5,6-epoxide were the major carotenoids, while chlorophyll *a* and *b'* were present in higher amounts. Frying of spinach leaves increased significantly the amount of α-tocopherol, β-carotene-5,6-epoxide, luteoxanthin, lutein, and its Z-isomers and chlorophyll *b'* isomer. There was significant decrease in the amounts of neoxanthin, violaxanthin, chlorophyll *b, b'* and chlorophyll *a* with increase of frying time. The increase of frying time increased the total phenolic contents in spinach leaves and fried sunflower oil samples. Chemical characteristics such as peroxide values, free fatty acids, conjugated dienes, conjugated trienes, and radical scavenging activity were significantly affected by frying, while spinach leaves increased the stability of the frying oil. This study can be used to improve the quality of fried vegetable leaves or their products at high temperature frying in food industries for increasing consumer acceptability.

## Introduction

Frying of foods is one of the traditional methods of food preparation. Frying is usually carried out in edible oils or fats as medium of choice. During frying triglycerides components of the oil or fats are oxidized at varying degree and are degraded (Aniolowska et al., 2016). The primary products formed are oxidized triglycerides (Zeb, 2015), which upon further oxidation produces secondary oxidation compounds such as various aldehydes and ketones. These oxidized products are toxic and have been found to negatively contributing to the quality of foods. However, it has been found recently that phenolic compounds present in the frying medium trap these toxic aldehyde and thus reduces toxicity (Zamora et al., 2016). It is thus important that some natural antioxidants should be present or may be retained in order to decrease toxicity during frying (Sunil et al., 2015). Both natural and synthetic antioxidants when heated at frying temperature could protect against the oxidative stress in the obese individuals (Perez-Herrera et al., 2013). Tomato products (Zeb and Haq, 2016), sea buckthorn products (Zeb and Ullah, 2015) and plant leaf extracts (Esposto et al., 2015) were used as good scavenging agents for decreasing toxic properties in *in vivo* or *in vitro* model systems. Vegetable leaves are of the special interest in food preparation.

Spinach (*Spinacia oleracea* L.) is a green leafy vegetable widely consumed in food preparation or as a food itself. Spinach contributes to several properties of the foods such as chemical, rheological, nutritional as well as sensory characteristics of the food (Khan et al., 2015). Spinach leaves are rich in carotenoids such as lutein and β-carotene. The composition of major carotenoids such as lutein ranges from 37 to 53 μg/kg, neoxanthin from 10 to 22 μg/kg, violaxanthin ranged from 9 to 23 μg/kg, while β-carotene contents are present in the range of 18–31 μg/kg (Bunea et al., 2008). *In vitro* digestion revealed an increase in the liberation of carotenoids especially lutein and β-carotene (Eriksen et al., 2016). Spinach powder was used as natural food grade antioxidants for the preparation in deep fried products (Lee et al., 2002). However, thermal treatment and high pressure produce changes in color and chemical attributes of an oil based spinach sauce (Medina-Meza et al., 2015). There is a lack of information about the changes in the carotenoids, chlorophylls, and its interactions with lipid oxidation during deep frying of vegetable leaves such as spinach. This study was therefore aimed for the first time to evaluate the interactions of lipid oxidation and changes in the pigment contents during frying of spinach leaves in sunflower oil.

## Materials and methods

### Chemicals and reagents

Lutein, β-carotene, chlorophyll a, BHT, and methanol were from Sigma-Aldrich (Steinheim, Germany). MTBE and ammonium acetate was purchased from Daejung Chemicals (Daejung, Korea). Ultrapure deionized double distilled water was prepared using Labtech distillation system (Namyangju, South Korea). All other chemicals and reagents were of analytical HPLC standard with highest purity. Luteoxanthin and mutatoxanthin standard was prepared from violaxanthin by acidification and subsequent TLC as reported previously (Gallardo-Guerrero et al., 2013). Similarly, lutein isomers were prepared using thermal stress and subsequent purification with TLC (Kimura and Rodriguez-Amaya, 2002).

### Sample collection and preparation

Indigenous fresh leaves of the *Spinacia oleracea* (oriental spinach) were collected from the local market in Chakdara, Khyber Pakhtunkhwa. The sample (1.0 kg) was randomly sampled from the open air marketed shelf. The latitude, longitude of sampling area is 34.65° and 72.033° and altitude of 2,288 ft. Fried and unfried spinach leaves were grinded during day light and control conditions to avoid further oxidation of carotenoid contents.

### Frying of leaves

The leaves were washed and cut into several pieces with dimension of 1 cm in length from all sides for the equal effects of heat processing. Representative samples of spinach leaves (200 g) were fried in sunflower oil (500 g) heated to the temperature of 250°C continuously for 15, 30, 45, and 60 min in laboratory open fryer. The unheated sample was taken as control. The selection of timing was based on the level of frying leaves in terms of visual color changes, while temperature was the one commonly used in Pakistan for street food frying, and mimicking the high temperature processing (pre-heating) in food industries (Weber et al., 2008). Each fried leaves and oil samples were collected in caped glass tubes and stored in refrigerator at −20°C till analysis.

### Extraction of carotenoids

Carotenoids extraction was carried out using the method from the recent work (Zeb, 2017). Sample processing and extractions were carried out under daylight, the mild nitrogen environment in a closed fume hood at 25°C, and in the absence of pressurized air, sunlight and high temperature. Briefly, one gram of fried and unfried grinded spinach paste was mixed with ice cold acetone (5 mL) and vortex for 60 min. Then 10 mL of absolute ice cold ethanol containing 0.1% BHT was added and again vortex for 30 min. The extractions were repeated until discoloration of spinach leaves. The solvent was evaporated under vacuum at 35°C. The residue was dissolved in to HPLC solvent (2 mL) and filtered using Agilent PFTE syringe filters (0.45 μm) and transferred into HPLC vials. Standard calibration curves of α-tocopherol, β-carotene epoxide, violaxanthin, lutein, its Z-isomers, and chlorophyll *a* were prepared for quantitative analyses. The limit of detection (LOD) and limit of quantification (LOQ) calculated from the standard curves were 0.4, 0.7, 0.9, 0.8, 0.05, 0.08, 0.31, 0.08 ng, and 2.5, 1.3, 1.6, 2.1, 1.1, 1.3, 2.0, 2.2 ng, for tocopherol, β-carotene epoxide, violaxanthin, luteoxanthin lutein, 9-Z-lutein, 9′-Z-lutein, and chlorophyll *a*, respectively.

### Chromatography

Carotenoids, chlorophylls and α-tocopherol were separated using a reversed phase HPLC system. The HPLC system (Agilent 1,260 Infinity Better) was equipped with quaternary pump, degasser, auto-sampler, and diode array detector. The column was an Agilent rapid resolution column (Agilent Zorbax Eclipse C18, Agilent Technologies, Waldbronn, Germany) with the specification of 4.6 × 100 mm, 3.5 μm maintained at 25°C. The tertiary gradient system consists of solvent A as methanol: deionized water (92: 8, v/v) with 10 mM ammonium acetate, solvent B was deionized water containing 0.01 mM and solvent C was MTBE (100%) (Zeb, 2017). The flow rate was fixed at 1 mL/min and injection volume was 50 μL. The gradient program was started with 80% A, 18% B, and 2% C. At 3 min the gradient was 80% A, 12% B and 8% C, which reached 65% A, 5% B with 30% C. The gradient then finally reached 60:0:40 (A:B:C)% at 40 min with post gradient elution of 10 min for recovery of the initial gradient. The spectra were recorded in the range of 190–750 nm and the chromatograms were obtained at 450 nm using OpenLab Chemstation software (Agilent Technologies, Germany). The identification of carotenoids, chlorophylls, and α-tocopherol were based on either the available standards, their retention times, or by comparing the absorption spectra reported in the literature. The identified compounds was quantified from the peak area using respective calibrations and represented as mg/100 g of the fresh weight basis.

### Lipid peroxidation

Lipid peroxidation was studied using a validated spectrophotometric method described recently (Zeb and Ullah, 2016) as thiobarbituric acid reactive substances (TBARS). Briefly lipid peroxidation products were extracted from the oil and leaves (1 g) samples using glacial acetic acid. The extract (1 mL) was mixed with 1 mL of thiobarbituric acid (TBA) reagent. The mixture was shaken for 1 h at 95°C. The absorbance of the room cooled mixture was measured at 532 nm in triplicates. The TBARS was measured as μmol/g of the sample.

### Chemical characteristics of oil

Chemical characteristics such as peroxide values, free fatty acids, conjugated dienes, and trienes of the fried and unfried sunflower oil samples were measured using standard AOCS methods (Firestone, 1998).

### Total phenolic contents

Extraction of phenolic compounds was carried out using methanol-water (50:50, v/v) from 1 g of the test sample. Total phenolic contents (TPC) of the oil and spinach leaf samples were measured using Folin-Ciocalteu (FC) reagent with optimized procedure as reported recently (Zeb and Rahman, 2017). Briefly, the sample extracts were mixed with 2 mL FC reagents and 2 mL Na_2_CO_3_ (7.5%) and incubated for 1 h. The absorbance of the samples were measured at 765 nm against the blank. The TPC was measured as mg of gallic acid equivalents (GAE) per 100 g of sample against the standard calibration curve of gallic acid.

### Data analyses

The data obtained was analyzed for statistical significance using one way analyses of variance with the help of Sigma-plot version 13.0.0 trial version 2014, (Systat Software, Inc., Chicago, Illinois, USA) for windows with *post hoc* Tukey test.

## Results and discussion

### Pigment composition of spinach leaves

**Figure 1:**
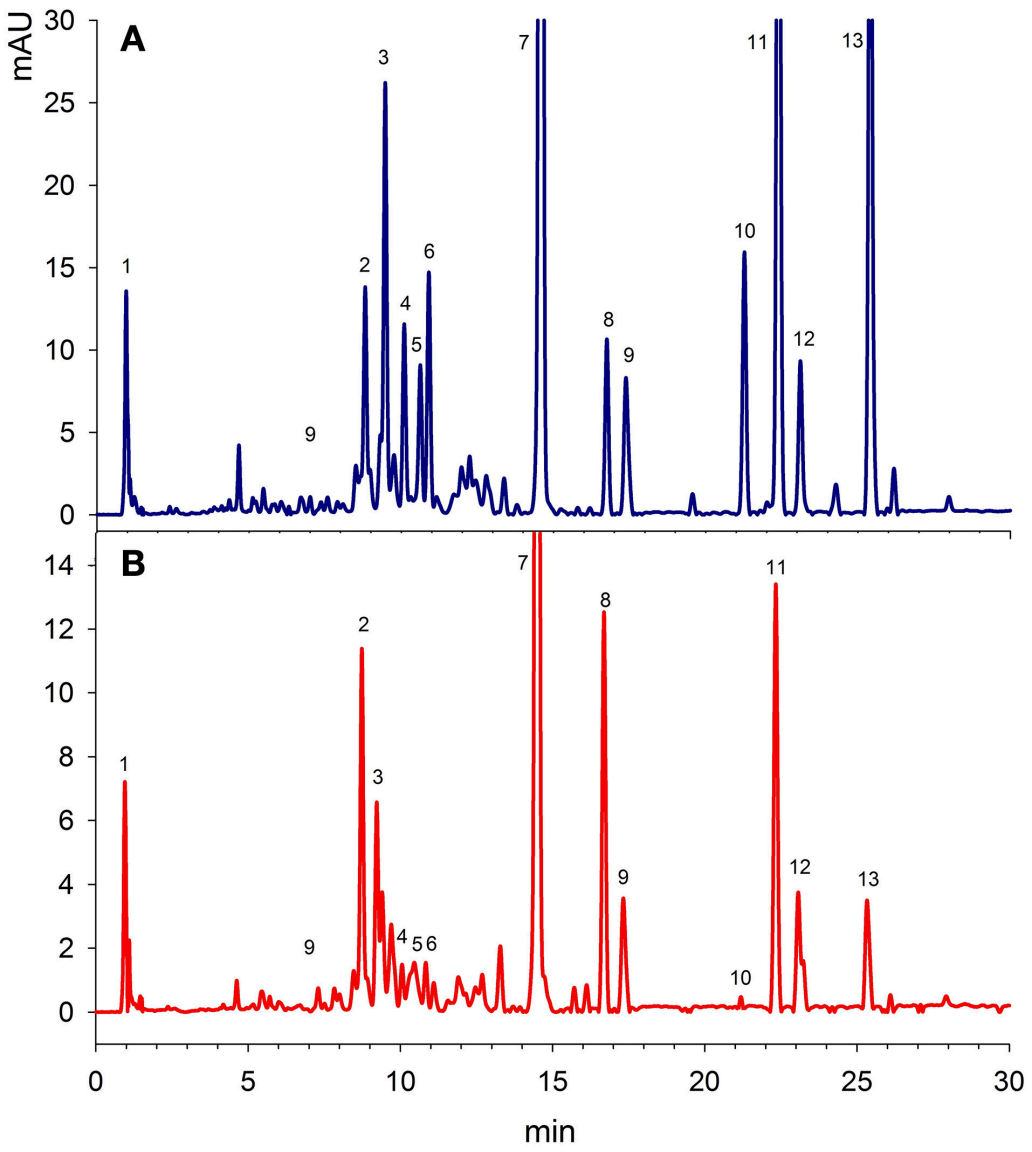
**A representative HPLC-DAD chromatograms of Spinach leaves at 450 nm. (A)** Unfried samples, **(B)** Fried samples of 60 min. The details identification is given at Table 1.

Figure 1 showed a good separation and identification of 13 pigment compounds with details as given in Table 1. Peak 1 was identified from the standard compound as α-tocopherol with λmax of 292 nm. Tang et al. (2005) showed that spinach leaves and vegetables supply significant amounts of tocopherol. Peak 2 was identified as β-carotene-5,6-epoxide from the standard compound prepared from the oxidation of β-carotene (Zeb, 2012). Peak 3 was found to be all-*E*-neoxanthin with λmax of 466, 436, and 416 nm. This compound was identified from the work of Minguez-Mosquera et al. (1992). Similarly, peak 4 was identified as all-*E*-violaxanthin λmax of 470, 440, and 416 nm from the standard compound. All-*E*-violaxanthin has strong photo-protective role in the leaf by transferring electron or energy from the chlorophylls to zeaxanthin (Pfündel and Bilger, 1994). The amount of violaxanthin was less (5.15 mg/100 g) than reported in a Belgium spinach variety (9–23 mg/100 g; Bunea et al., 2008). Luteoxanthin was identified as peak 5 with λmax of 448, 422, and 398 nm and elution time of 10.9 min. Compound 6 was found to be mutatoxanthin with λmax of 452, 424, and 400 nm. Lutein was eluted at the retention time of 14.5 min with characteristics λmax of 472, 446, and 422 nm. The amount of lutein was higher (19.1 mg/100 g) than reported recently in the Slovenian spinach variety (5–15 mg/100 g; Simonovska et al., 2013). Two 9-trans isomers of lutein i.e., 9-Z-lutein and 9′-Z-lutein were also present in <2 mg/100 g and were identified as peak 8 and 9 respectively. Peak 10–13 were chlorophyll *b, b*' and its isomer and chlorophyll *a*. Among the chlorophylls, a high amount (19.3 mg/100 g) of chlorophyll *a* was present, followed by chlorophyll *b*' with concentration of 15.6 mg/100 g. Chlorophylls are photo-synthetic pigments and are present in large amounts in vegetable leaves. The results of the present study reported a lower amounts of total chlorophylls in Indian spinach leaves than reported by Singh (2016). The author showed that phytochemical level in the spinach can be increased by foliar spraying of chitosan treatment. Thus, chitosan treatment, variety, location and uses of direct analysis in real time mass spectrometry (DART-MS) may be the reasons of difference in the phytochemical composition. It was suggested that the real time mass spectrometry analyses can provide a plus point to this study, which will help to determine the exact structure of chlorophyll isomer and several un-identified compounds detected.

**Table 1:** **Identification of carotenoids in Spinach leaves (oriental)**.

**Rt**	**Identity**	**Absorption spectra**	%III/II^*^	**References**
0.9	α-Tocopherol	292	–	Standard
8.8	β-Carotene-5,6-epoxide	470, 444, 420	29.5	Standard
9.4	All-*E*-neoxanthin	466, 436, 416	84.6	Minguez-Mosquera et al., 1992
10	All-*E*-violaxanthin	470, 440, 416	86.5	Standard
10.6	All-*E*-luteoxanthin	448, 422, 398	96.1	Standard
10.9	All-*E*-mutatoxanthin	452, 424, 400	94.0	Minguez-Mosquera et al., 1992
14.5	All-*E*-Lutein	472, 446, 422	66.9	Standard
16.7	9-Z-lutein	468, 440, 418	71.3	Standard
17.3	9'-Z-lutein	466, 438, 418, 330	32.5	Standard
21.2	Chlorophyll b	648, 460	–	Minguez-Mosquera et al., 1992
22.3	Chlorophyll b'	648, 464	–	Minguez-Mosquera et al., 1992
23.1	Chlorophyll b' isomer	648, 462	–	Minguez-Mosquera et al., 1992
25.4	Chlorophyll a	664, 432	–	Standard

*The compounds were identified by comparing absorption spectra of the sample with the standard compounds or from the values reported in the literature*.

* *The %III/II is the percent ratio of the height of the absorption peak of longest-wavelength, designated III, and that of the middle absorption peak, designated II, taking the minimum between the two peaks as baseline. This ratio represent the spectral fine structure of carotenoid*.

### Effects of frying on carotenoids contents

Table 2 shows that significant (*p* < 0.01) increase occurred in the amounts of α-tocopherol with an increase in heat treatment time. Tocopherol is a strong natural antioxidant, which stabilizes frying oils (Aladedunye, 2014). The increase stability of sunflower oil may be due to the presence of tocopherol in spinach leaves as well as in the sunflower oil. Similarly, a significant (*p* < 0.01) increase was also observed in the concentration of β-carotene-5,6-epoxide from 1.44 to 6.64 mg/100 g of the control to 60 min of heat treatment. The formation of this epoxide is a well-known phenomenon during thermal treatment of β-carotene (Zeb, 2012). Epoxy carotenoids are more stable than carotenes during thermal stress model system (Hadjal et al., 2013). The increase in β-carotene-5,6-epoxide may thus be due to stability and increased formation from β-carotene. All-*E*-neoxanthin (2.62 mg/100 g) increased significantly (*p* < 0.05) at 15 min of thermal treatment and significantly reduced to 1.27 mg/100 g during 30 and remained stable till 45 min of treatment. A significant increase occurred and reached to 2.18 mg/100 g during 60 min of treatment. All-*E*-violaxanthin decreased significantly (*p* < 0.01) from 5.15 to 0.78 mg/100 g at control and 60 min treatment respectively. The decrease of violaxanthin was highly correlated with increase of luteoxanthin amounts. Similarly, mutatoxanthin increased at 15 min, but was found to decline significantly (*p* < 0.01) with increase of thermal treatment.

**Table 2:** **Effects of frying on the carotenoids, chlorophylls, and α-tocopherol contents in Spinach leaves in sunflower oil at 250°C**.

**Peak**	**Compounds**		**Amount (mg/100 g)**				
		***P* < value**	**Control**	**15**	**30**	**45**	**60**
1	α-tocopherol	0.01	0.73 ± 0.05^a^	1.91 ± 0.11^b^	2.87 ± 0.16^c^	3.18 ± 0.05^d^	3.64 ± 0.12^e^
2	β-Carotene-5,6-epoxide	0.01	1.44 ± 0.05^a^	2.91 ± 0.13^b^	1.33 ± 0.11^a^	4.65 ± 0.08^c^	6.65 ± 0.25^d^
3	All-*E*-Neoxanthin	0.05	2.62 ± 0.15^a^	4.78 ± 0.21^b^	1.27 ± 0.09^c^	1.53 ± 0.11^c^	2.18 ± 0.15^d^
4	All-*E*-Violaxanthin	0.01	5.15 ± 0.21^a^	2.24 ± 0.12^b^	1.79 ± 0.08^c^	1.83 ± 0.06^c^	0.78 ± 0.13^d^
5	Luteoxanthin	0.01	0.42 ± 0.05^a^	1.83 ± 0.03^b^	1.52 ± 0.08^c^	1.12 ± 0.11^d^	2.13 ± 0.04^e^
6	Mutatoxanthin	0.01	0.58 ± 0.04^a^	2.71 ± 0.11^b^	0.46 ± 0.02^c^	0.63 ± 0.03^a^	0.83 ± 0.02^d^
7	Lutein	0.001	19.1 ± 1.13^a^	37.5 ± 0.95^b^	44.2 ± 1.21^c^	47.8 ± 1.14^d^	50.3 ± 1.31^d^
8	9-*Z*-Lutein	0.01	10.1 ± 0.05^a^	1.99 ± 0.16^b^	4.68 ± 0.21^c^	5.11 ± 0.11^d^	7.49 ± 0.33^e^
9	9'-*Z*-Lutein	0.01	2.84 ± 0.04^a^	2.01 ± 0.02^b^	2.45 ± 0.05^c^	2.52 ± 0.5^c^	1.37 ± 0.06^d^
10	Chlorophyll b	0.01	2.11 ± 0.01^a^	3.14 ± 0.03^b^	2.01 ± 0.13^a^	0.13 ± 0.01^c^	0.11 ± 0.03^c^
11	Chlorophyll b'	0.05	15.6 ± 0.91^a^	13.9 ± 0.45^b^	11.3 ± 0.45^c^	9.93 ± 0.53^d^	8.45 ± 0.31^e^
12	Chlorophyll b' isomer	0.05	2.61 ± 0.01^a^	2.05 ± 0.01^b^	2.74 ± 0.02^c^	2.92 ± 0.04^d^	3.61 ± 0.05^e^
13	Chlorophyll a	0.01	19.3 ± 1.01^a^	10.3 ± 0.72^b^	5.11 ± 0.31^c^	4.0 ± 0.31^d^	2.82 ± 0.31^e^

*Values are expressed as mean ± SD of n = 3 readings. Different letter (a-e) in the same row represent significant at the given p-values*.

All-*E*-lutein was 19.1 mg/100 g and significantly (*p* < 0.001) increased to 37.5, 44.2, 47.8, and 50.0 mg/100 g corresponding to 15, 30, 45, and 60 min of thermal treatment respectively. These results are in contrast to the results of Arnold et al. (2014), who showed that heat treatment reduced lutein contents in spinach leaves. This may be due to the liberation of lutein from protein or lipid bound form to free lutein, as in the current work, the separation has not detected exactly the ester of lutein. It was also possible that lutein from oil medium enters the leave matrix and contributed to the increased amounts. Eriksen et al. (2016) showed that *in vitro* digestion of spinach liberate carotenoids especially lutein and β-carotene. Similarly, the 9-Z-isomers of lutein also increased significantly (*p* < 0.01) with increase of thermal treatment. This confirms the hypothesis that lutein from bound form contributed to the unbound as well its isomer formation.

The amount of chlorophyll *b* and *b*' decreased significantly with increase of thermal treatment. Chlorophyll *b* initially increased to 3.14 mg/100 g from its control values of 2.1 mg/100 g and then reduced significantly (*p* < 0.01) to 0.13 mg/100 g at 60 min of thermal treatment. Chlorophyll *b*' isomers decreased initially to 2.05 mg/100 g at 15 min from 2.61 mg/100 g of the control values and reached 3.61 mg/100 g at 60 min of thermal treatment. There is a positive correlation between the increase of chlorophyll *b*' isomers and decrease of chlorophyll *b* and *b*' contents, which suggests their degradation to its chlorophyll *b*' isomer. Previous studies (Teng and Chen, 1999) showed that pyrochlorophylls and their derivatives were produced during heating. Thus, it is possible that the isomer of chlorophylls may be pyrochlorophyll, which need further investigation using mass spectrometry to confirm.

### Effects of frying on total phenolic contents

**Figure 2:**
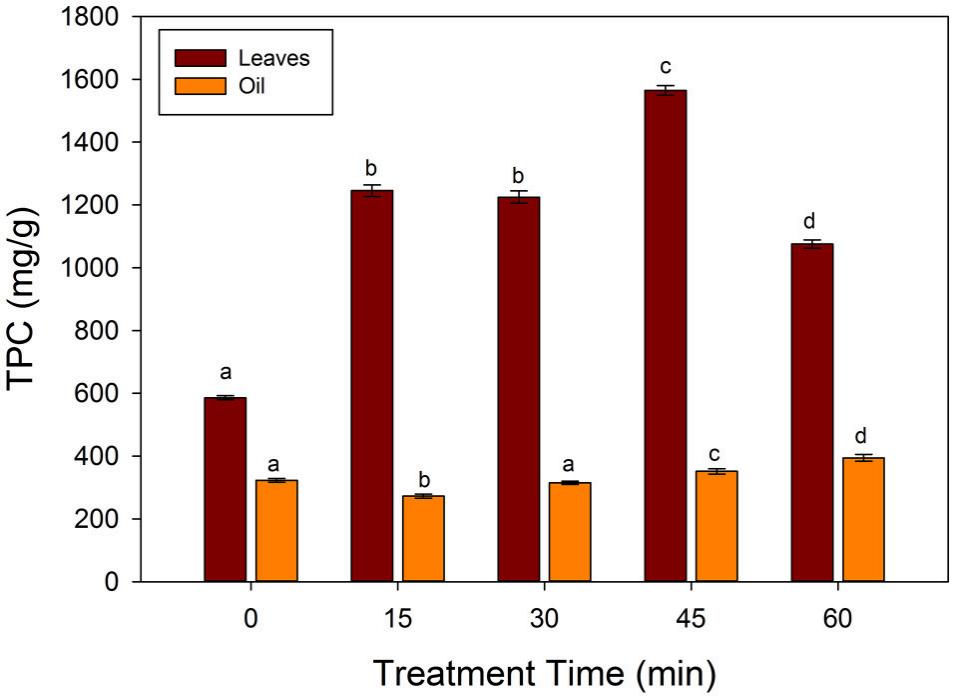
**Changes in the total phenolic contents (TPC) during frying of spinach leaves at 250°C in sunflower oil**. Different letters (a–d) in the same bars represents significance at (*p* < 0.05) of *n* = 5. Values are expressed as mean ± standard deviation (SD) of replicate readings. The error bars in each column represent its corresponding SD values.

Figure 2 showed the changes in the total phenolic contents of frying oil and spinach leaves fried at 250°C. The TPC of the unfried spinach leaves was 585.6 mg/g of GAE, which increased significantly (*p* < 0.05) till 45 min of frying, while a significant decrease was observed at 60 min (1075.5 mg/g GAE). In case of frying oil, TPC increased significantly at 45 and 60 of frying, however, the amount of TPC in oil was very small as compared to the spinach leaves. In a study of Turkmen et al. (2005), the TPC in the spinach showed an increase retention with different cooking methods. The initial increase may be due to the liberation of phenolic compounds from the leave matrix. The decrease of TPC at 60 min of frying may be due to the degradation of phenolic compounds as well as leaching to the frying medium. Ismail et al. (2004), showed that TPC significantly decreased in the water boiled spinach, cabbage, kale and shallots leaves. The authors suggested that the degradation of phenolic compounds during boiling may be reason of loss in TPC. In case of sunflower oil, the TPC decreased significantly (*p* < 0.05) at 15 min and increased further with increase of frying time. The increase in the TPC was highly correlated (*R*^2^ = 0.8435) with a decrease in the TBARS values of the sunflower oil. The present results are in agreement with the Silva et al. (2001), who showed that the addition of phenolic compounds increased the stability of sunflower oil. These authors showed that caffeic acid derivatives showed an increase in the stability of sunflower oil than α-tocopherol. The present results indicate that during frying of spinach leaves, increase in the TPC occurred in the sunflower oil, which may be responsible for the increase in stability and decrease in the lipid peroxidation. Thus, spinach or spinach containing products may contribute to the increase in the stability of frying oils.

### Effects of frying on chemical characteristics of oil

**Figure 3:**
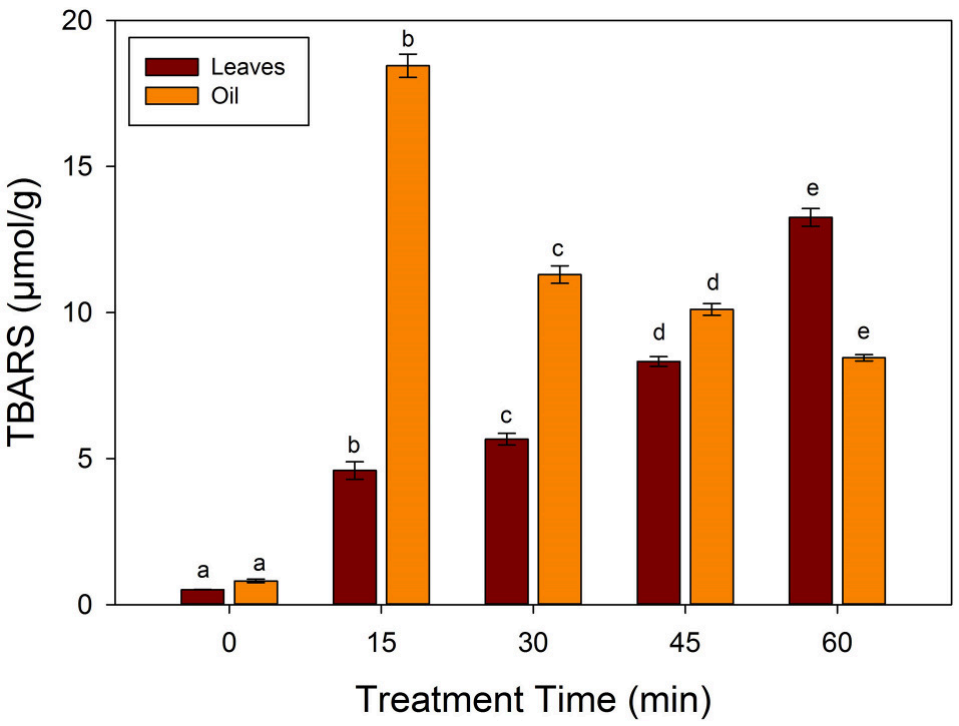
**Changes in the TBARS values during frying of spinach leaves at 250°C in sunflower oil**. Different letters (a-e) in the same bars represents significance at (*p* < 0.05) of *n* = 5. Values are expressed as mean ± SD of replicate readings. The error bars in each column represent its corresponding SD-values.

Lipid peroxidation was determined using TBARS produced during frying with values as shown in the Figure 3. Sunflower oil and spinach leaves have a TBARS values of 0.81 and 0.51 μmol/g respectively. A significant increase was observed in the TBARS values at 15 min of frying at 250°C to 18.45 and 4.59 μmol/g in oil and spinach leaves respectively. A significant (*p* < 0.05) increase occurred in the TBARS values in the fried leaf samples, which is in accordance with the reported literature that thermal treatment increase TBARS contents (Domínguez et al., 2014). The strongest correlation in the TBARS values with increasing frying time was a clear indicator that TBARS contents of the oil was assimilated in the leaf matrix. Thus, it is important to minimize the TBARS contents in the fried foods.

**Figure 4:**
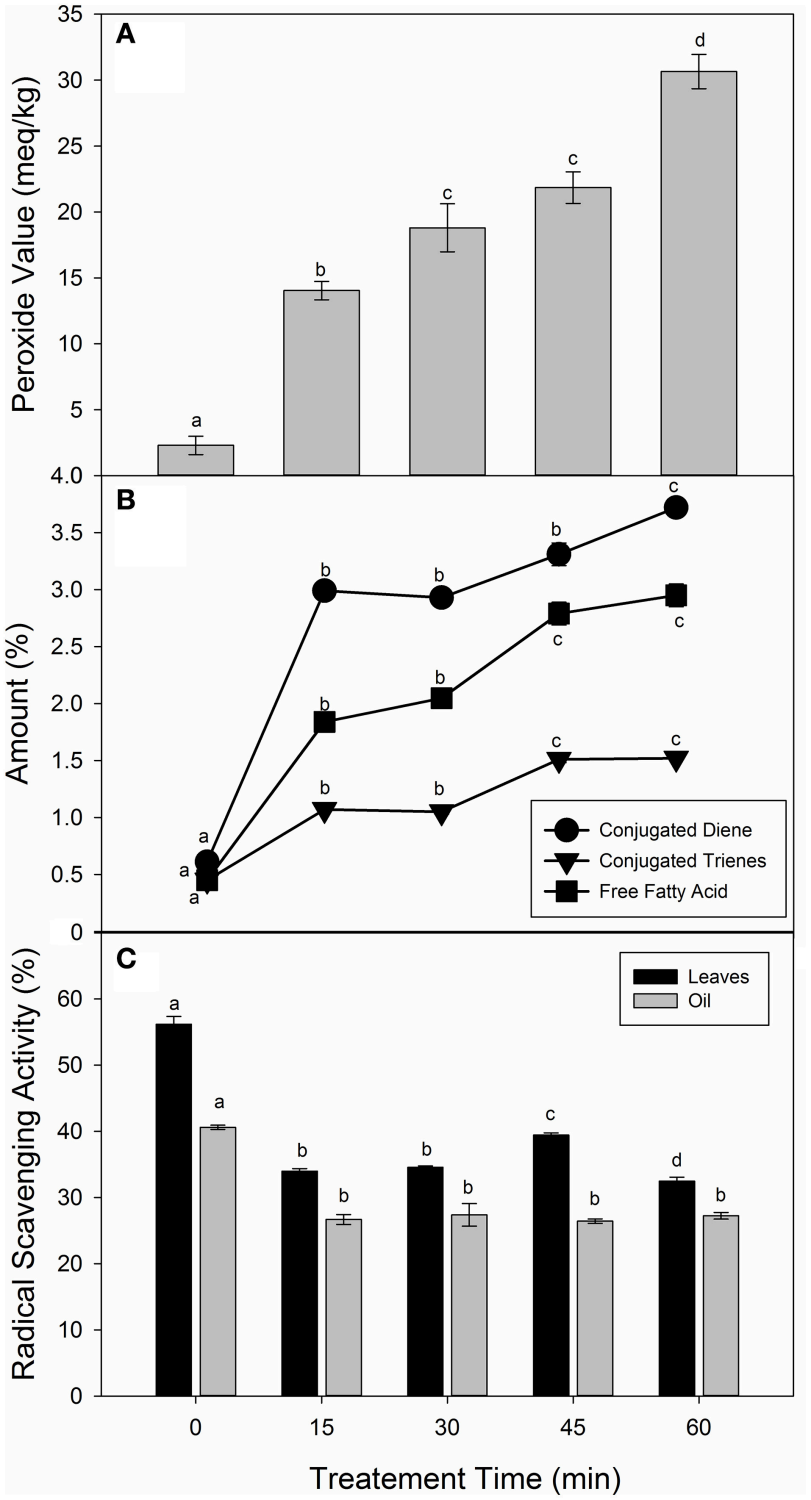
**Changes in the chemical characteristics of sunflower oil during frying of spinach leaves at 250°C. (A)** Peroxide value of oils, **(B)** Conjugated dienes, trienes and free fatty acids of oils, and **(C)** Radical scavenging activity. Different letters (a-d) in the same bars represents significance at (*p* < 0.05) of *n* = 5 measurements. Values are expressed as mean ± SD of replicate readings. The error bars in each column represent its corresponding SD-values.

In order to understand the interactions of lipid oxidation during frying of vegetable leaves, it is imperative to evaluate the chemical characteristics of frying oil. Four quality parameters, i.e., peroxide values (PV), free fatty acids (FFA), conjugated dienes (CD) and trienes values (CT) of the frying oils were determined. Figure 4A showed the changes in the PV of the sunflower oil during frying of the spinach leaves at 250°C. Peroxide values increased significantly (*p* < 0.05) of the control (2.30 meq/kg) to 30.65 meq/kg of the 60 min of frying. There was no significant (*p* > 0.05) difference between the PV-values of 30 and 45 min of frying. The results are in agreement with the previous findings (Zeb and Murkovic, 2013; Ramadan, 2015). The non-significant changes during 30 and 45 min may be due to the increased availability of carotenoids and phenolic compounds in the system. The increase of TBARS in the leaves samples was highly correlated (*R*^2^ = 0.9599) with the increase in the peroxide values of oil. This suggests an accumulation of oxidized species of triacylglycerols such as hydro-peroxides, hydroxides, and epoxides in fried oil, enters the spinach leaves matrix and oxidized further to form secondary lipid peroxidation products.

Figure 4B showed that changes in the CD, CT, and FFA-values of fried sunflower oil samples. The CD-values increased significantly at 15 min of frying with no significant difference till 45 min of frying. A significant increase in the CD-values of the sunflower oil was obtained at 60 min of frying. Similar observations were also reported in the CT and FFA-values. Ramadan (2013) showed that CD and CT-values of sunflower oil increased with increase frying time, which was also the case in our work. The non-significant difference in the CD and CT-values may be due to the presence of natural antioxidants in the sunflower oil and spinach leaves. Natural antioxidants from the plant sources were good scavengers of free radicals that stabilize FFA, CD and CT-values of fats and oils (Aladedunye, 2014). This suggests that antioxidants such as carotenoids and tocopherol (Table 1) in the spinach leaves contributed toward the control formation of secondary lipid peroxidation products in sunflower oil during frying.

Figure 4C revealed the changes in the radical scavenging activity (RSA) of the spinach leaves and sunflower oil samples. The RSA-values of the spinach leaves showed a significant (*p* < 0.05) decrease with respect to the increase in the frying time. There was a significant (*p* < 0.05) decrease in the RSA-values after frying at 15 min, but had no significant effects on increasing the frying time. The non-significant effects of frying time may be due to the increase in the phenolic and carotenoid contents, which are leached to the sunflower oil during frying. The addition of phenolic compounds or carotenoids extend the stability of the oil toward thermal stress (Esposto et al., 2015). These results indicated for the first time, that spinach leaves when fried in sunflower oil at high temperature, the stability of the oil increased due to the leaching of phenolic compounds and carotenoids. Thus, foods products comprising of spinach and edible oil when prepared at relatively high temperature may retain important phytochemicals.

## Conclusions

The study presented the effects of high temperature frying of spinach leaves on the carotenoids, chlorophylls and α-tocopherol for the first time. Spinach leaves revealed eight carotenoids, four chlorophylls and α-tocopherol. Lutein, neoxanthin, violaxanthin and β-carotene-5,6-epoxide, chlorophyll *a* and *b'* were present in higher amounts. Frying of spinach leaves increased significantly the amount of α-tocopherol, β-carotene-5,6-epoxide, luteoxanthin, lutein, and its Z-isomers and chlorophyll *b'* isomer. Significant decrease in the amounts of neoxanthin, violaxanthin, chlorophyll *b, b'* and chlorophyll *a* occurred with the increase of frying time. The increase of frying time increased the total phenolic contents in spinach leaves and fried sunflower oil samples. Chemical characteristics of sunflower oil such as peroxide, free fatty acid, conjugated dienes, conjugated trienes, and radical scavenging activity were significantly affected by frying and the presence of spinach leaves had a positive role in oil stability. This study can be used to improve the quality of fried vegetable leaves or their products at high temperature frying in food industries for consumer acceptability.

## Author contributions

The work is part of the project financed to AZ, who designed the experiments, carried out HPLC analysis and interpretation, PN collected samples, did rest of the experiments, AZ interpret results and wrote the paper.

## Funding

The authors are highly grateful for the financial assistant by the Higher Education Commission (HEC) Pakistan under National Research Program for Universities (NRPU), project No. 2344.

### Conflict of interest statement

The authors declare that the research was conducted in the absence of any commercial or financial relationships that could be construed as a potential conflict of interest.
